# Incident Cognitive Impairment During Aging in Rural South Africa: Evidence From the HAALSI Cohort, 2014 to 2019

**DOI:** 10.1093/geroni/igaa057.511

**Published:** 2020-12-16

**Authors:** Lindsay Kobayashi, Meagan Farrell, Kenneth Langa, Nomsa Mahlahela, Ryan Wagner, Lisa Berkman

**Affiliations:** 1 University of Michigan, Ann Arbor, Michigan, United States; 2 Harvard T. H. Chan School of Public Health, Cambridge, Massachusetts, United States; 3 University of the Witwatersrand, Acornhoek, Mpumalanga, South Africa

## Abstract

We estimated the incidence of cognitive impairment and its key sociodemographic, social, and health-related predictors at the first longitudinal follow-up of the population-representative “Health and Aging in Africa: A Longitudinal Study of an INDEPTH Community in South Africa” (HAALSI) cohort of adults aged ≥40 in rural Agincourt, South Africa. Cognitive impairment was defined as scoring ≥1.5 SD below the baseline mean composite time orientation and episodic memory score, or requiring a proxy interview with “fair” or “poor” proxy-reported memory. Activity of daily living (ADL) limitations were compared according to incident cognitive impairment status. Incidence rates (IRs) and rate ratios (IRRs) for cognitive impairment according to sociodemographic, social, and health-related predictors were estimated using Poisson regression with robust standard errors, and weighted to account for mortality. Over a 3.7-year mean follow-up, 309/3,861 at-risk participants newly developed cognitive impairment (IR=24.0/1000 person-years (PY); 95% CI: 21.6-26.8). Incidence increased from IR=9.1/1000 PY (95% CI: 5.5-16.1) among those aged 40-44 at baseline to IR=76.5/1000 PY (95% CI: 63.2-93.4) among those aged 80+. At least one ADL limitation was prevalent in 39% of those with incident cognitive impairment, compared to 7% of non-impaired participants. Incident cognitive impairment did not vary by sex/gender, HIV status, or cardiovascular factors, but was strongly graded according to education, literacy, household assets, employment, marital status, and frequency of alcohol consumption. This study presents one of the first incidence rate estimates for cognitive impairment in sub-Saharan Africa. Social disparities in cognitive impairment were apparent in patterns similar to many high-income countries.

